# Recycling Thermoset Systems by Vitrimerization Using Solid‐State Shear Extrusion‐ A Feasibility Study

**DOI:** 10.1002/gch2.202500417

**Published:** 2025-12-04

**Authors:** Amin Jamei‐Oskouei, Majid Mehrabi‐Mazidi, Ica Manas‐Zloczower

**Affiliations:** ^1^ Department of Macromolecular Science and Engineering Case Western Reserve University Cleveland Ohio USA

**Keywords:** dynamic covalent network, epoxy recycling, mechanochemistry, solid‐state shear extrusion, vitrimerization

## Abstract

Thermoset polymers with permanently cross‐linked networks have outstanding mechanical properties but cannot be reprocessed or recycled. Vitrimerization is a simple and practical method to convert permanent crosslinked thermosets into vitrimers with covalent adaptable networks, which can be recycled. Vitrimerization is a mechanochemical strategy to convert thermosets into vitrimers by using a ball milling system. In this study, we propose solid‐state shear extrusion (SSSE) as a continuous, room‐temperature route to replace ball milling (BM) for epoxy vitrimerization. The vitrimerized thermosets obtained using the SSSE process exhibit comparable activation energy and mechanical properties with the vitrimers obtained using the BM method. In addition, the SSSE vitrimers can be reprocessed multiple times, maintaining above 80 percent in mechanical properties. This first feasibility study of employing SSSE for vitrimerization may establish it as a scalable, energy‐efficient alternative to batch BM for industrial, closed‐loop recycling of thermosets with the least environmental impact.

## Graphical abstract

Vitrimerization converts permanent thermoset networks into dynamic covalent ones (vitrimers) that can be reshaped and recycled. Solid‐state shear vitrimerization via extrusion (SSSE) offers a continuous, ambient‐temperature alternative to high‐energy batch ball milling: metal‐carboxylates ligands formed during SSSE serve as junctions for the transesterification exchange reaction during subsequent compression molding, building a robust, recyclable vitrimer network.

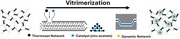

## 1 Introduction

Thermoset polymers, which account for approximately 18% of all polymers produced globally, have an estimated annual production volume of over 65 million tons [1]. Their exceptional thermal stability, mechanical strength, and dimensional rigidity make them indispensable in high‐performance applications such as aerospace, automotive, wind energy, electronics, and coatings [2]. However, their permanent covalent crosslinked networks render them infusible and insoluble, posing significant challenges for end‐of‐life management. As a result, the vast majority of thermoset waste is still handled through landfilling, incineration, or mechanical grinding into low‐value fillers [3]. Current recycling methods, such as pyrolysis and solvolysis, are either energy‐intensive, environmentally harmful, and unable to recover the original polymer matrix [2, 3]. These limitations have led to recycling rates for thermosets that remain drastically low compared to thermoplastics. Consequently, there is an urgent need for innovative and scalable recycling technologies that can preserve or regenerate the structural integrity of thermoset matrices while aligning with circular economy principles. Vitrimerization, the conversion of permanent thermoset networks into dynamic covalent structures, offers a pathway to reprocessing and recycling these materials by enabling reversible bond exchange reactions under heat [4, 5, 6, 7, 8, 9, 10, 11, 12].

Our research group has pioneered vitrimerization as a mechanochemical recycling strategy for various crosslinked polymers, including epoxy systems [5, 6, 7, 13], unsaturated polyester resins [8], polyurethane foams [9], crosslinked poly (ethylene‐vinyl acetate) elastomers [10, 11], and thermosets containing carbonate and thiourethane linkages [12]. Previous efforts have predominantly employed ball milling or cryogenic ball milling with liquid nitrogen. These techniques not only pulverized thermosets into fine particles, enhancing reactive surface area, but also facilitated uniform catalyst dispersion and promoted ligand formation near dynamic functional groups to enable efficient bond exchange during reprocessing.

While effective, batch‐wise ball milling limits throughput, scalability, and continuity. To address these challenges, this study explores solid‐state shear extrusion (SSSE) using a twin‐screw extruder as a continuous mechanochemical approach to recycle thermoset polymers. Extrusion, a widely used technique for the melt‐processing of thermoplastics, typically involves high‐temperature flow and shaping of materials [14, 15]. However, under certain conditions, extrusion can also be applied in the solid state, a technique known as solid‐state shear extrusion (SSSE) or solid‐state shear pulverization (SSSP) [16, 17]. SSSE or SSSP, originally developed for polymer powder production, polymer compatibilization, and additive incorporation, applies high shear and compressive forces at sub‐melting temperatures. This process not only reduces particle size but also drives mechanochemical interactions such as in situ compatibilization and chain scission [18, 19, 20, 21, 22].

Applying SSSE to vitrimerization offers four primary advantages:

1. Particle Size Reduction: Increases reactive surface area for bond exchange reactions.
2. Energy‐Driven Catalyst Incorporation: Facilitates catalyst dispersion and complexation with dynamic functional groups.
3. Continuous Processing: Provides scalability beyond batch ball milling.
4. Processing at Ambient Temperature: Saves more energy and is more efficient.

**FIGURE 1:**
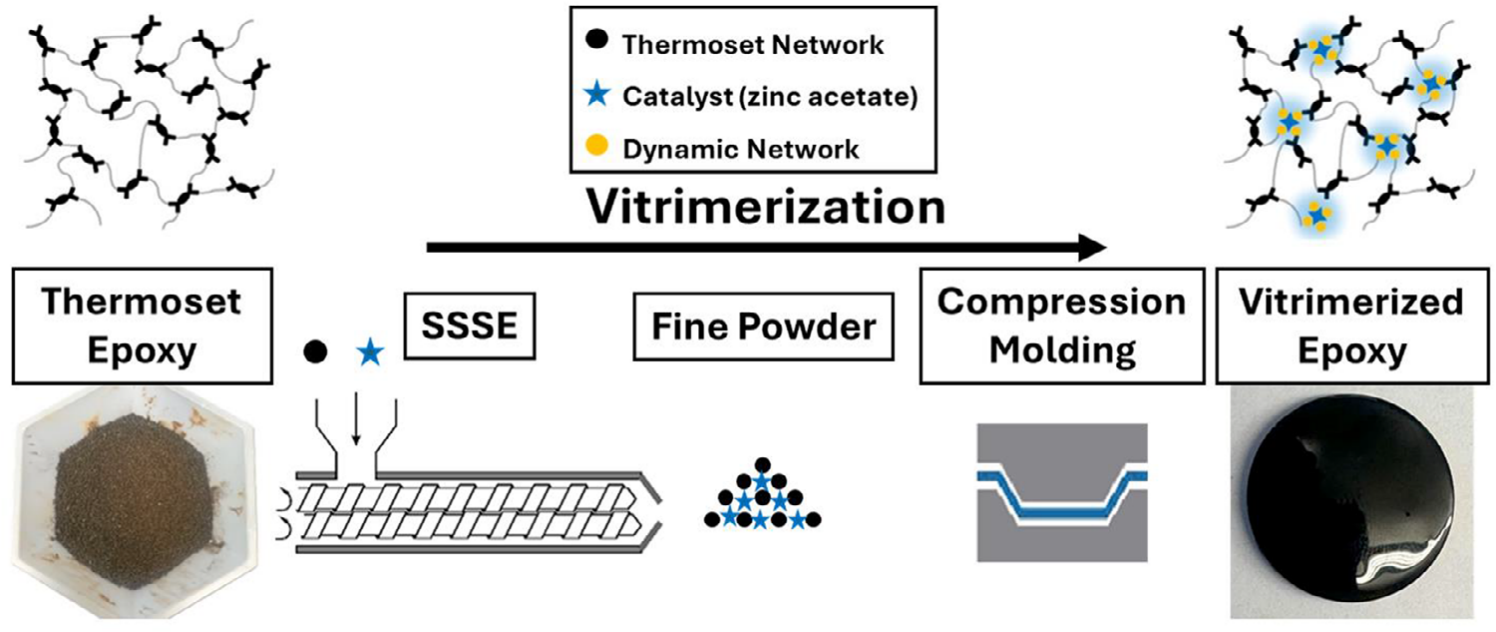
Schematic illustration of vitrimerization via solid‐state shear extrusion (SSSE) and subsequent compression molding to form a reprocessable vitrimer network.

In this feasibility study, we demonstrate for the first time the use of a twin‐screw extruder to achieve vitrimerization of a crosslinked epoxy thermoset through an SSSE process (Figure 1). This continuous, scalable method leverages mechanochemical principles to incorporate a catalyst directly into the crosslinked network at ambient temperature, in a solvent‐free and lower‐hazard approach relative to chemical/thermal recycling routes. Using zinc acetate (Zn(OAc)_2_), a non‐toxic, commercially available catalyst, we successfully recycle permanently crosslinked epoxy by enabling dynamic covalent exchanges via transesterification. Unlike previous studies that relied on batch ball milling (BM), which requires repeated loading and unloading cycles, this approach enables continuous vitrimerization, paving the way for practical industrial implementation of thermoset recycling. Although in the present study a lab‐scale twin‐screw extruder has been used, requiring manual collection of extrudate, the SSSE process itself is inherently continuous. To evaluate the effectiveness of this process, we benchmarked the performance of SSSE against our previously established BM method, comparing chemical structure, dynamic behavior, and mechanical properties of the resulting vitrimer networks.

## 2 Results and Discussion

**FIGURE 2:**
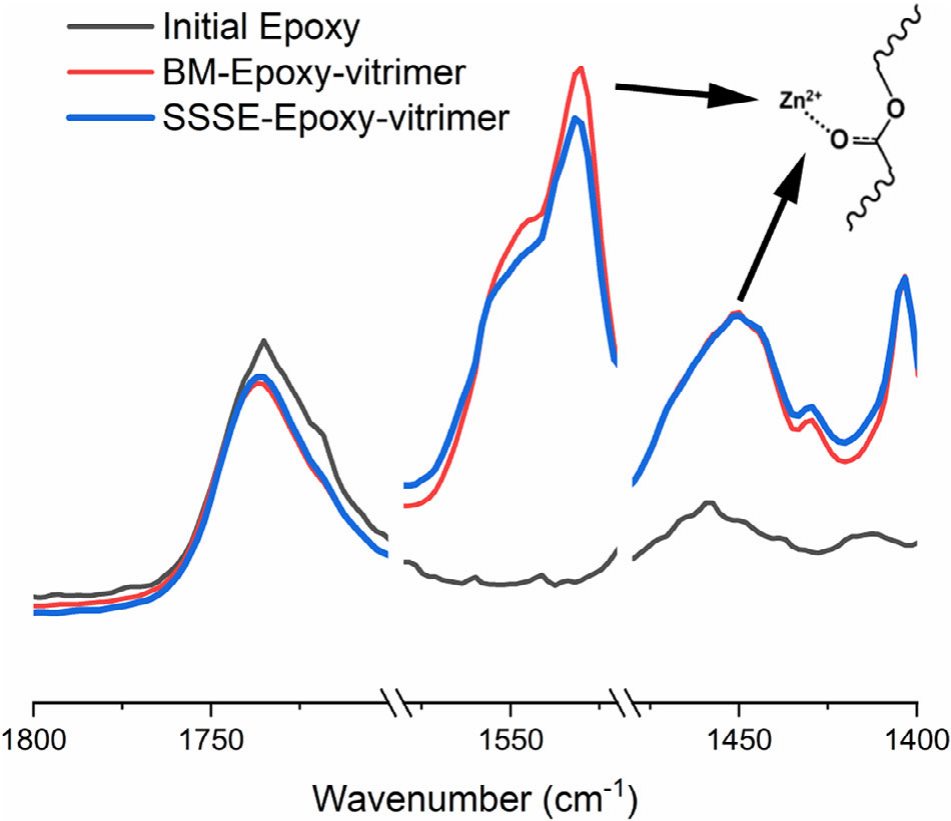
The Fourier transform infrared (FTIR) spectra of the initial epoxy and vitrimerized epoxies containing 10 parts per hundred resin (phr) of zinc acetate (Zn(OAc)2), processed through BM and SSSE, reveal significant spectral changes. Notably, the intensities of the carbonyl absorption bands, located between 1760 and 1690 cm^−1^, exhibit a decrease, while the vibrations associated with carboxylate‐zinc complexes, observed at 1520−1560 cm^−1^ and 1406 cm^−1^, show an increase following the vitrimerization process.

The mechanochemical vitrimerization process involves the conversion of permanently crosslinked networks into dynamic networks using a catalyst. For epoxy vitrimerization, a critical initial step involves the formation of zinc–carboxylate complexes, where zinc ions coordinate with the carbonyl groups of ester functionalities. These complexes not only facilitate transesterification reactions but also contribute to the formation of dynamic covalent cross‐links within the vitrimer network, enabling adaptive behavior [5, 23, 24]. To verify this ligand formation, Fourier Transform Infrared (FTIR) spectroscopy tests were conducted, and the results are presented in Figure 2 (the full‐width spectra have been displayed in Figure S1). The curves represent the average intensities of four FTIR tests for each sample. The intensity of the carbonyl groups between 1760 and 1690 cm^−1^ exhibits a slight decrease following vitrimerization, accompanied by the emergence of new peaks at 1406 cm^−1^ and within the range of 1520–1560 cm^−1^. These peaks are indicative of the successful formation of zinc ligands with carbonyl groups in epoxy [5].

**FIGURE 3:**
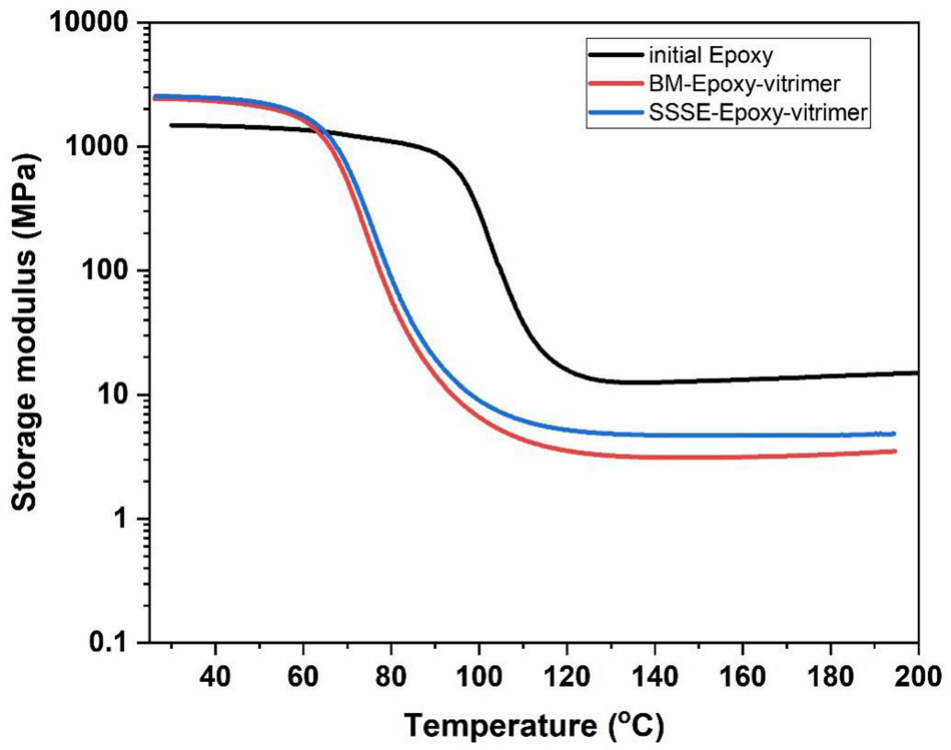
Storage modulus (E′) as a function of temperature for initial epoxy, BM epoxy vitrimer, and SSSE epoxy vitrimer. Both vitrimerized samples show increased modulus at room temperature and a comparable *T_g_* region, validating the structural integrity of the dynamic networks and similar thermomechanical properties post‐vitrimerization.

**FIGURE 4:**
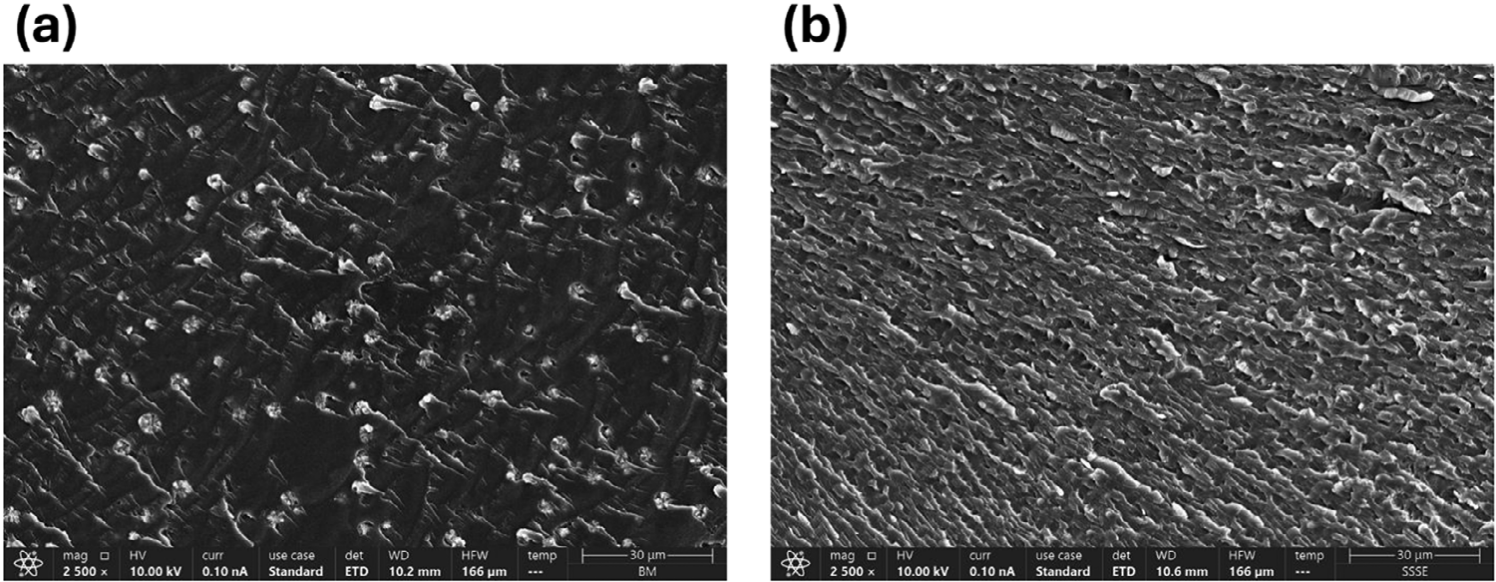
SEM micrographs of cross‐sections from the (a) BM‐Epoxy‐vitrimer and (b) SSSE‐Epoxy‐vitrimer. Zn‐containing domains are visible in the BM‐Epoxy‐vitrimer sample. Better dispersion of the catalyst in the SSSE‐Epoxy‐vitrimer is observed.

The thermomechanical performance of the samples was evaluated using DMA temperature sweep tests (Figure 3). Both vitrimerized samples, i.e., BM and SSSE, showed a notable increase in storage modulus at room temperature compared to the initial epoxy (i.e., from 1.48 GPa in epoxy to 2.46 GPa in vitrimerized samples). This increase in stiffness is attributed to the presence of zinc acetate, which plays a dual role in the system. A portion of the zinc acetate forms coordination complexes with ester groups, resulting in zinc–carboxylate ligands that may contribute to network reinforcement and localized rigidity [5, 25, 26]. In parallel, the unreacted or excess zinc acetate particles may remain dispersed within the matrix, effectively acting as inorganic fillers and further enhancing stiffness. Figure 4 presents SEM micrographs of cross‐sections from the BM‐Epoxy‐vitrimer and SSSE‐Epoxy‐vitrimer. Discrete Zn‐containing domains are visible in the BM‐Epoxy‐vitrimer sample; however, a much finer dispersion of the catalyst particles is apparent for the SSSE‐Epoxy‐vitrimer sample. At the magnifications used, the particle–matrix interfaces appear well bonded (no observable interfacial debonding), which is consistent with a modest, filler‐like contribution to the stiffness. Similar upward trends in stiffness with catalyst loading have also been reported previously for vitrimerized zinc‐catalyzed epoxy [4, 5]. This combination of chemical coordination and physical reinforcement offers a plausible explanation for the increased storage modulus observed at room temperature in vitrimerized samples. Both vitrimerization routes lowered the glass‐transition temperature (*T*_g_) (Figure S2), consistent with a reduction in cross‐link density (Table S1). Additionally, the transition behavior near the *T_g_* remained consistent between the BM and SSSE samples, indicating that the vitrimerization method did not significantly alter the thermal transition of the material.

These results demonstrate that SSSE can produce vitrimerized materials with mechanical and thermal characteristics comparable to those achieved via ball milling.

**FIGURE 5:**
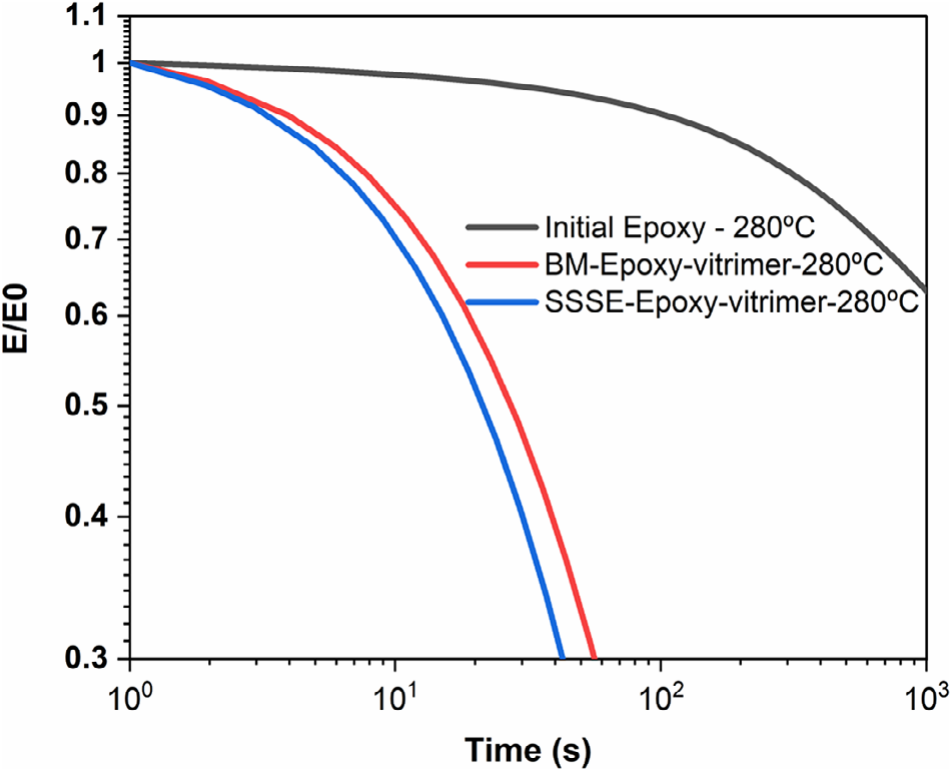
Normalized relaxation modulus over time at 280°C showing the stress relaxation behavior for initial epoxy, BM epoxy vitrimer, and SSSE epoxy vitrimer. While the initial epoxy shows limited relaxation, both vitrimerized samples exhibit rapid stress decay, confirming effective dynamic bond exchange and vitrimer‐like behavior at elevated temperature.

Stress relaxation tests conducted at 280°C (Figure 5) revealed that both BM‐ and SSSE‐ vitrimerized samples showed rapid stress relaxation, characteristic of dynamic bond exchange. In contrast, the initial epoxy did not show appreciable stress relaxation under identical conditions, reaffirming the permanent nature of the original thermoset network.

Both vitrimerized samples achieved near‐complete relaxation within similar timescales, confirming that SSSE is equally capable of introducing dynamic bonds necessary for vitrimer formation, despite its continuous and short processing time compared to the batch‐ball milling procedure.

To evaluate the temperature‐dependent relaxation behavior, stress relaxation tests were performed at multiple temperatures ranging from 200°C to 280°C. The resulting data were fitted using the stretched exponential decay, Kohlrausch–Williams–Watts (KWW) equation (Figures S3 and S4) [27, 28, 29], which provides two parameters: the characteristic relaxation time (τ*) and the stretch exponent (β), reflecting the distribution of relaxation times. To enable direct comparison, the average relaxation time ⟨τ*⟩ was calculated using the following Equation (1):

(1) $$\langle \tau^{*}\rangle =\tau^{*}\times \frac{\Gamma \left(\frac{1}{\beta }\right)}{\beta }$$

where *Γ* is the gamma function. The calculated average relaxation times for both BM and SSSE samples are summarized in Tables S2 and S3, respectively.

**FIGURE 6:**
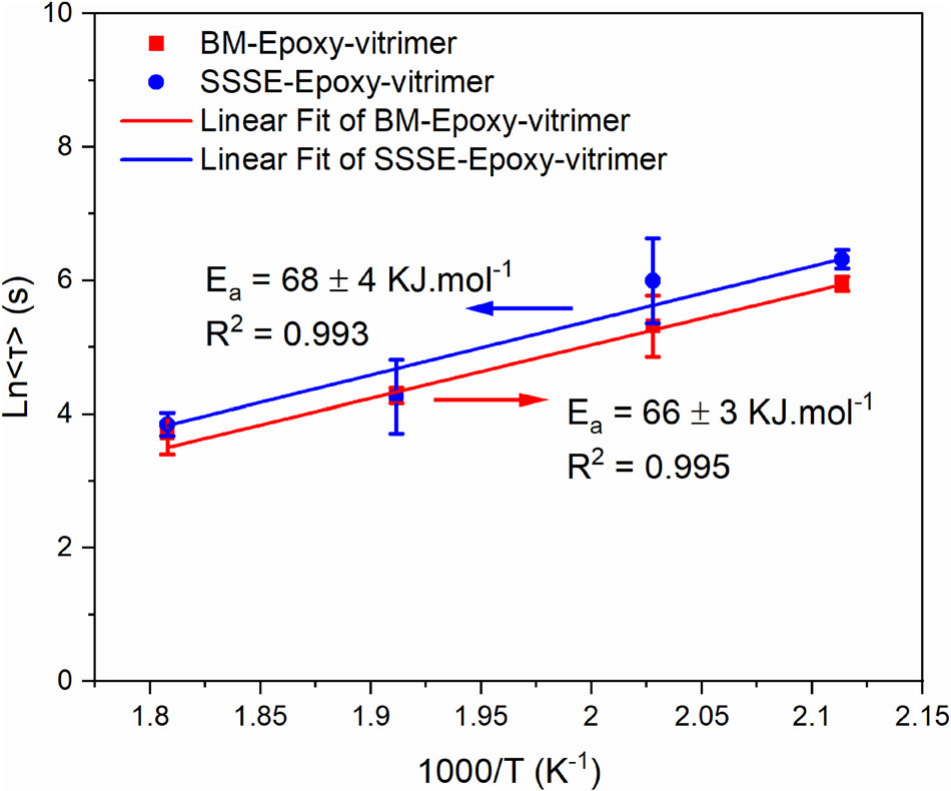
Arrhenius plots constructed from average relaxation times determined using KWW fitting for BM and SSSE epoxy vitrimers. Both samples display linear temperature dependence, with activation energies of 66 ± 3 kJ.mol^−1^ (BM) and 68 ± 4 kJ.mol^−1^ (SSSE), consistent with transesterification dynamics and successful vitrimerization.

Using these values, Arrhenius plots (Figure 6) were constructed to determine the apparent activation energy (E_a_) for the relaxation process, following Equation (2):

(2) $$\langle \tau^{*}\rangle =\langle \tau_{0}^{*}\rangle \exp\left\{\frac{E_{a}}{\mathrm{RT}}\right\}$$

The fitted activation energies were 66 ± 3 kJ.mol^−1^ for the BM vitrimerized epoxy and 68 ± 4 kJ.mol^−1^ for the SSSE vitrimerized epoxy. Both values are statistically indistinguishable within their error limits and fall within the expected range for catalyst‐assisted transesterification reactions [27, 30, 31], thereby supporting the successful formation of dynamic covalent networks in both vitrimerization pathways.

Mechanical performance, a key metric for practical applicability, was assessed through tensile testing (Figures S5–S10). The tabulated data can be found in Table 1. The vitrimerized samples exhibited a higher Young's modulus than the initial epoxy, suggesting that vitrimerization results in a stiffer material. This finding is in line with the higher storage modulus of the vitrimers compared to that of the original epoxy in the glassy region, as discussed above. Vitrimerization led to a moderate (∼30%–40%) decrease in ultimate tensile strength compared to the initial epoxy. Comparing the vitrimerized epoxies, the sample prepared by SSSE process exhibited a higher elastic modulus and tensile strength than the BM‐processed sample. The reason for the superior tensile properties of the SSSE vitrimer by comparison with the BM vitrimer originates from the difference in the shape, size, and size distribution of the epoxy particles obtained during these two processes. It should be noted that the vitrimerization process, through milling and compression molding of the resulting powders at elevated temperatures and under high pressure, is strongly controlled by welding as well as the exchange reactions between the micronized epoxy particles during molding [5]. The welding and packing of the particles are strongly influenced by the size and geometry of the particles produced during milling and grinding. As shown in Figure S11a–d, both BM and SSSE processes generate epoxy powders with a bimodal size distribution; however, the size distribution in the SSSE process is much narrower than in the BM process. In other words, the particles obtained by the BM process contain a significant proportion of coarse particles (above 500 µm), whereas such coarse particles are not present in the SSSE The coarse particles have a small surface area, and their presence in the epoxy sample reduces the interfacial surface area between the particles during the welding and vitrimerization process under compression molding. Thus, the level of interfacial welding and exchange reactions can be adversely affected. As a result, lower ultimate tensile strength is expected for the epoxy vitrimers made of coarser particles. In addition, the coarse particles can act as stress concentration sites in the material during mechanical loading, further reducing the strength of the resulting vitrimer sample. Thus, the ultimate tensile strength of the SSSE vitrimer is greater than that of the BM vitrimer.

**TABLE 1:** Tabulated data of tensile properties for initial epoxy, BM‐Epoxy‐vitirmer, SSSE‐Epoxy‐vitirmer, and reprocessed SSSE‐Epoxy‐vitirmer samples.

Sample code name	Young's modulus (MPa)	Ultimate tensile strength (MPa)	Elongation at break (%)
Initial epoxy	1070 ± 152	51.4 ± 4.7	8.4 ± 3.8
BM‐epoxy‐vitrimer	1293 ± 189	30.2 ± 2.0	2.7 ± 1.4
SSSE‐epoxy‐vitrimer	1400 ± 120	34.3 ± 2.7	2.1 ± 0.5
SSSE‐epoxy‐vitrimer‐ 1^st^ rep.	1350 ± 67	30.8 ± 2.8	2.5 ± 0.5
SSSE‐epoxy‐vitrimer‐ 2^nd^ rep.	1320 ± 100	30.0 ± 1.5	2.5 ± 0.5
SSSE‐epoxy‐vitrimer‐ 3^rd^ rep.	1300 ± 140	28.8 ± 1.5	2.9 ± 0.3

**FIGURE 7:**
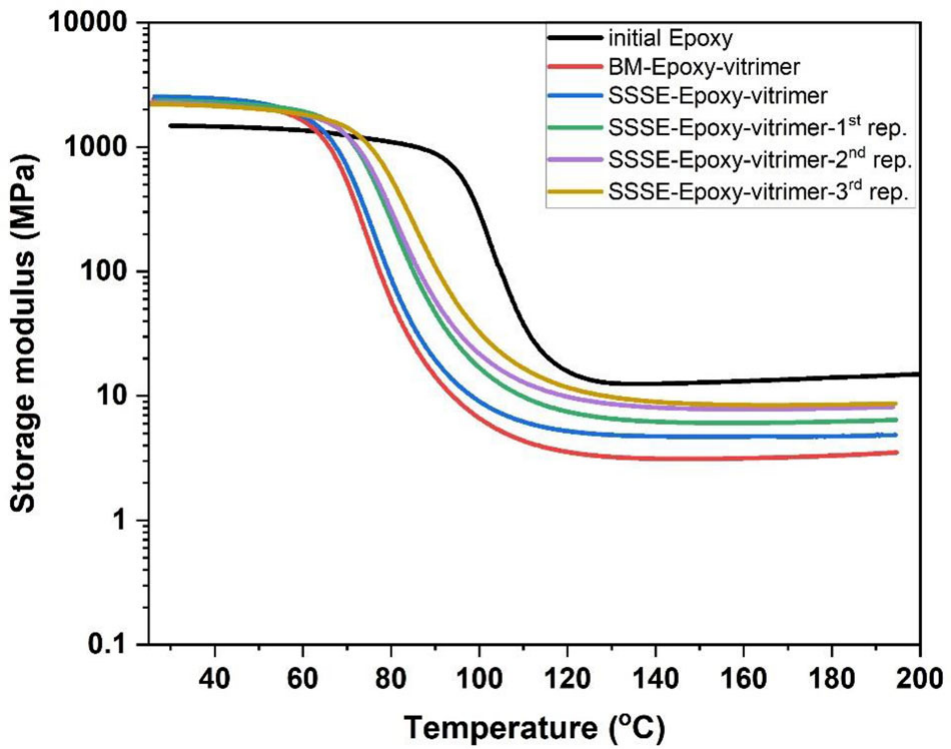
Temperature dependence of storage modulus for initial epoxy, BM‐epoxy‐vitrimer, SSSE‐Epoxy‐vitrimer, and reprocessed SSSE vitrimers.

To assess reprocessability, the SSSE epoxy vitrimers were reprocessed three times via consecutive grinding and compression molding and evaluated through both tensile (Figures S8–S10) and thermomechanical (DMA) analyses (Figure 7). The elastic modulus remained almost unchanged after three reprocessing cycles; however, the ultimate tensile strength slightly decreases upon reprocessing (Table 1).

Interestingly, the thermomechanical behavior, as captured by DMA reveals that the reprocessed samples maintain a slightly higher glass transition temperatures and even exhibit slightly higher storage modulus values in the rubbery plateau region at elevated temperatures compared to the initial SSSE vitrimer (Figure 7). This increase in elasticity above *T_g_* could probably stem from potential additional local crosslinking during compression molding. Furthermore, the formation of free radicals followed by their recombination during each reprocessing step may contribute to a gradual increase in *T_g_* and rubbery plateau [5].

It should be pointed out that the ultimate tensile strength in the vitrimer samples prepared in this work depends not only on the degree of crosslinking but also on epoxy particles packing, catalyst particles’ size and size distribution, as well as the dispersion and distribution of catalyst particles in the matrix. Any change in these microstructural features, as a result of successive reprocessing steps (grinding and remolding), can have a significant effect on the ultimate tensile strength of the resulting vitrimers, regardless of the trend of *T*_g_ in the DMA test.

Overall, these results demonstrate that the vitrimerized sample produced via SSSE can be successfully reprocessed with minimal property loss, especially when processed under controlled conditions. It should be noted that this work represents a feasibility study designed to demonstrate the comparative potential of continuous SSSE versus batch ball milling; detailed process optimization and broader formulations screening will be addressed in future work.

## 3 Conclusions

In summary, we demonstrate the successful vitrimerization of epoxy‐anhydride thermosets through solid‐state shear extrusion (SSSE), introducing a continuous and scalable alternative to the conventional batch ball milling method. Using a lab‐scale twin‐screw extruder, we achieved effective and fast pulverization as well as catalyst incorporation at ambient temperature, enabling the formation of dynamic covalent networks via transesterification. The vitrimerized samples processed using SSSE showed comparable thermomechanical properties, stress relaxation behavior, and activation energy to the sample processed by ball milling, confirming the efficacy of the continuous method. FTIR results indicated the successful formation of zinc–carboxylate complexes, while DMA and stress relaxation tests verified the dynamic behavior of the resulting vitrimers. Mechanical testing revealed that the SSSE samples exhibit a higher Young's modulus compared to the BM one. Additionally, reprocessing of SSSE samples confirmed recyclability, with only slight reductions in mechanical performance. Overall, this feasibility study advances the vitrimerization technology by replacing a batch route with a continuous extrusion‐based process, offering a more practical path toward industrial implementation of thermoset recycling. Further optimization of the extrusion conditions and post‐processing steps may enhance the vitrimers' properties even further, especially at larger scales.

## 4 Experimental Section

### 4.1 Materials

Zinc acetate, bisphenol A diglycidyl ether (DGEBA), glutaric anhydride, and imidazole were purchased from Sigma‐Aldrich.

### 4.2 Preparation of Epoxy Anhydride Thermoset

DGEBA (1 eq. epoxy groups), glutaric anhydride (0.75 epoxy equivalents), and imidazole (3 wt.% to DGEBA) were homogeneously mixed and cured at 100°C for 4 h, following a post‐curing at 160°C for another 4 h to ensure complete crosslinking [5, 6, 32, 33].

### 4.3 Vitrimerization of Epoxy Anhydride Thermoset by Ball Milling (BM)

To benchmark the vitrimerization process, according to our previous work [5], the cured epoxy (10 g) was first ground into small particles by using a high‐speed grinder for a few minutes (∼1 mm particles). This size was chosen to enable consistent feeding into the lab‐scale ball milling jar. Then the ground epoxy was ball milled (Fritsch pulverisette 6) with 10 phr zinc acetate for 1 h at a speed of 600 rpm, yielding fine powder. The powder sample was compression‐molded at 250°C and 5 MPa for 1 h. Samples processed via ball milling were identified with the prefix BM.

### 4.4 Vitrimerization of Epoxy Anhydride Thermoset by Solid‐State Shear Extrusion (SSSE)

Vitrimerization of the ground epoxy‐anhydride thermoset (ground to ∼ 1 mm by using a high‐speed grinder to enable consistent feeding into the lab‐scale twin‐screw extruder) was performed using a Process 11 twin‐screw extruder (ThermoFisher Scientific, USA) operated at ambient temperature under solid‐state conditions in less than a minute. To facilitate both pulverization of the cured thermoset and incorporation of the zinc acetate catalyst, a dual‐feeder setup was employed to ensure a constant catalyst loading of 10 parts per hundred resin (phr). The material was processed through the extruder three times to enhance homogenization; while the majority of particle size reduction occurred during the first pass, the subsequent passes primarily improved catalyst dispersion and overall mixture uniformity. The resulting powder was then subjected to compression molding at 250°C under a pressure of 5 MPa for 1 h. Samples processed via solid‐state shear extrusion were identified with the prefix SSSE.

### 4.5 Reprocessing of Vitrimerized Epoxy Anhydride

Fractured specimens obtained from tensile testing were further broken into mm‐sized pieces and reprocessed to evaluate the material's recyclability. The fragments were placed in a stainless‐steel compression mold, sandwiched between two plates, and subjected to molding at 250°C under 5 MPa pressure for 1 h to ensure complete filling and consolidation.

### 4.6 Characterizations

*Fourier transform infrared spectroscopy (FTIR)*: FTIR analyses were carried out using an Agilent Cary 630 in a spectral range of 4000–650 cm^−1^.

*Dynamic Mechanical Analysis (DMA)*: TA Instruments Q800, operating in tensile mode with a constant frequency of 1 Hz, strain amplitude of 0.05%, was employed for a temperature sweep test to determine the storage modulus (Eʹ) and glass transition temperature (Tg) (determined from the peak of the tan δ) by scanning at 5°C/min from 25°C to 180°C. The samples were rectangular with a thickness of 1–1.5 mm, a length of 25 mm, and a width of 5–6 mm.

*Stress Relaxation Measurements*: Stress relaxation experiments were carried out with the TA Instruments Q800, operating in tensile mode with a constant strain of 1% under a nitrogen atmosphere on rectangular samples with a thickness of 1–1.5 mm, a length of 25 mm, and a width of 5–6 mm. Three independent specimens were tested at each temperature to demonstrate replicability.

*Mechanical testing*: Stress–strain curves were obtained on an Instron 1011 universal testing instrument in tensile mode. The samples were specimens 60 mm × 12 mm × 1.5 mm, and the strain rate was 5 mm/min. Multiple tests were conducted for each sample to guarantee replicability.

*Thermogravimetric Analysis (TGA)*: The thermal stability was studied by TGA using TA Instruments Q500 with an aluminum pan. The samples were about 10 mg each and were run from room temperature to 600°C at a heating rate of 10°C/minute under constant N2 flow.

*Scanning electron microscopy (SEM)*: ThermoFisher Apreo2 SEM was used to characterize the morphology and dispersion of zinc acetate in the fracture surface.

*Optical Microscopy*: Optical micrograms were taken by Keyence VHX‐5000 to visually show the size distribution of the BM‐ and SSSE‐ powders and the cross‐section of molded tensile specimens for BM and SSSE vitrimers.

## Conflicts of Interest

The authors declare no conflicts of interest.

## Supporting information




**Supporting file**: gch270075‐sup‐0001‐SuppMat.docx

## Data Availability

The data that support the findings of this study are available from the corresponding author upon reasonable request.

## References

[1] S. Ma and D. C. Webster , “Degradable Thermosets Based on Labile Bonds or Linkages: A Review,” Progress in Polymer Science 76 (2018): 65–110, 10.1016/j.progpolymsci.2017.07.008.

[2] Y. Liu , Z. Yu , B. Wang , P. Li , J. Zhu , and S. Ma , “Closed‐Loop Chemical Recycling of Thermosetting Polymers and Their Applications: A Review,” Green Chemistry 24, no. 15 (2022): 5691–5708, 10.1039/D2GC00368F.

[3] W. Post , A. Susa , R. Blaauw , K. Molenveld , and R. J. I. Knoop , “A Review on the Potential and Limitations of Recyclable Thermosets for Structural Applications,” Polymer Reviews 60, no. 2 (2020): 359–388, 10.1080/15583724.2019.1673406.

[4] L. Yue , V. S. Bonab , D. Yuan , A. Patel , V. Karimkhani , and I. Manas‐Zloczower , “Vitrimerization: A Novel Concept to Reprocess and Recycle Thermoset Waste via Dynamic Chemistry,” Global Challenges 3, no. 7 (2019): 1800076, 10.1002/gch2.201800076. 31565382 PMC6607417

[5] L. Yue , H. Guo , A. Kennedy , et al., “Vitrimerization: Converting Thermoset Polymers Into Vitrimers,” ACS Macro Letters 9, no. 6 (2020): 836–842, 10.1021/acsmacrolett.0c00299. 35648515

[6] L. Yue , M. Amirkhosravi , X. Gong , T. G. Gray , and I. Manas‐Zloczower , “Recycling Epoxy by Vitrimerization: Influence of an Initial Thermoset Chemical Structure,” ACS Sustainable Chemistry & Engineering 8, no. 33 (2020): 12706–12712, 10.1021/acssuschemeng.0c04815.

[7] L. Yue , M. Amirkhosravi , K. Ke , T. G. Gray , and I. Manas‐Zloczower , “Cellulose Nanocrystals: Accelerator and Reinforcing Filler for Epoxy Vitrimerization,” ACS Applied Materials Interfaces 13, no. 2 (2021): 3419–3425, 10.1021/acsami.0c19350. 33412839

[8] A. Bandegi , M. Amirkhosravi , H. Meng , M. K. R. Aghjeh , and I. Manas‐Zloczower , “Vitrimerization of Crosslinked Unsaturated Polyester Resins: A Mechanochemical Approach to Recycle and Reprocess Thermosets,” Global Challenges 6, no. 7 (2022): 2200036, 10.1002/gch2.202200036. 35860393 PMC9284659

[9] A. Bandegi , M. Montemayor , and I. Manas‐Zloczower , “Vitrimerization of Rigid Thermoset Polyurethane Foams: A Mechanochemical Method to Recycle and Reprocess Thermosets,” Polymers for Advanced Techs 33, no. 10 (2022): 3750–3758, 10.1002/pat.5827.

[10] A. Bandegi , T. G. Gray , S. Mitchell , et al., “Vitrimerization of Crosslinked Elastomers: A Mechanochemical Approach for Recycling Thermoset Polymers,” Materials Advances 4, no. 12 (2023): 2648–2658, 10.1039/D3MA00098B.

[11] A. J. Oskouei , E. Mao , T. G. Gray , et al., “Vitrimerization of Crosslinked Poly(Ethylene‐Vinyl Acetate): The Effect of Catalysts,” RSC Applied Polymers 2, no. 5 (2024): 905–913, 10.1039/D4LP00112E.

[12] R. Rahimzadeh , Y. Han , and I. Manas‐Zloczower , “A Mechanochemical Approach to Recycle Thermosets Containing Carbonate and Thiourethane Linkages,” Polymer 298 (2024): 126877, 10.1016/j.polymer.2024.126877.

[13] L. Yue , K. Ke , M. Amirkhosravi , T. G. Gray , and I. Manas‐Zloczower , “Catalyst‐Free Mechanochemical Recycling of Biobased Epoxy with Cellulose Nanocrystals,” ACS Applied Bio Materials 4, no. 5 (2021): 4176–4183, 10.1021/acsabm.0c01670. 35006830

[14] N. Shaghaghi , E. Steinmetz , T. Schneider , and J. Maia , “Burst Pressure Performance of Multilayer Co‐Extruded Polystyrene/Poly(Methyl Methacrylate) Pipes,” Polymer Engineering and Science 65 (2025): 4061–4069, 10.1002/pen.27270.

[15] C. Rauwendaal , R. Gonzalez‐Nunez , and D. Rodrigue , “Polymer Processing: Extrusion,” Encyclopedia of Polymer Science and Technology (John Wiley & Sons, Ltd, 2017), 1–67, 10.1002/0471440264.pst126.pub2.

[16] K. Khait and S. H. Carr , Solid‐State Shear Pulverization, A New Polymer Processing and Powder Technology (CRC Press, 2001).

[17] K. Khait and J. M. Torkelson , “Solid‐State Shear Pulverization of Plastics: A Green Recycling Process,” Polymer‐Plastics Technology and Engineering 38, no. 3 (1999): 445–457, 10.1080/03602559909351592.

[18] E. Bilgili , H. Arastoopour , and B. Bernstein , “Pulverization of rubber granulates using the solid‐state shear extrusion (SSSE) Process:: Part I. Process Concepts and Characteristics,” Powder Technology 115, no. 3 (2001): 265–276, 10.1016/S0032-5910(00)00353-3.

[19] P. J. Brunner , J. T. Clark , J. M. Torkelson , and K. Wakabayashi , “Processing‐structure‐property relationships in solid‐state shear pulverization: Parametric study of specific energy,” Polymer Engineering & Science 52, no. 7 (2012): 1555–1564, 10.1002/pen.23115.

[20] T. A. Will , Y. Lu , and K. Wakabayashi , “Effects of Polymer Properties on Solid‐State Shear Pulverization: Thermoplastic Processability and Nanofiller Dispersibility,” ACS Applied Polymer Materials 5, no. 3 (2023): 1848–1858, 10.1021/acsapm.2c01932. 36968143 PMC10034747

[21] K. A. Iyer and J. M. Torkelson , “Sustainable Green Hybrids of Polyolefins and Lignin Yield Major Improvements in Mechanical Properties When Prepared via Solid‐State Shear Pulverization,” ACS Sustainable Chemistry & Engineering 3, no. 5 (2015): 959–968, 10.1021/acssuschemeng.5b00099.

[22] M. Ganglani , J. M. Torkelson , S. H. Carr , and K. Khait , “Trace Levels of Mechanochemical Effects in Pulverized Polyolefins,” Journal of Applied Polymer Science 80, no. 4 (2001): 671–679, 10.1002/1097-4628(20010425)80:4<671::AID-APP1143>3.0.CO;2-B.

[23] M. Capelot , M. M. Unterlass , F. Tournilhac , and L. Leibler , “Catalytic Control of the Vitrimer Glass Transition,” ACS Macro Letters 1, no. 7 (2012): 789–792, 10.1021/mz300239f. 35607118

[24] A. Demongeot , S. J. Mougnier , S. Okada , C. Soulié‐Ziakovic , and F. Tournilhac , “Coordination and Catalysis of Zn 2+ in Epoxy‐Based Vitrimers,” Polymer Chemistry 7, no. 27 (2016): 4486–4493, 10.1039/C6PY00752J.

[25] Y. Wang , Z. Liu , C. Zhou , et al., “A Facile Strategy for High Performance Recyclable Polymer Systems via Dynamic Metal Ion Crosslinking,” Journal of Materials Chemistry A 7, no. 8 (2019): 3577–3582, 10.1039/C8TA11866C.

[26] X. Niu , F. Wang , X. Kui , et al., “Dual Cross‐linked Vinyl Vitrimer With Efficient Self‐Catalysis Achieving Triple‐Shape‐Memory Properties,” Macromolecular Rapid Communications 40, no. 19 (2019): 1900313, 10.1002/marc.201900313. 31393644

[27] F. Cuminet , D. Berne , S. Lemouzy , et al., “Catalyst‐Free Transesterification Vitrimers: Activation via α‐Difluoroesters,” Polymer Chemistry 13, no. 18 (2022): 2651–2658, 10.1039/D2PY00124A.

[28] T. Isogai and M. Hayashi , “Critical Effects of Branch Numbers at the Cross‐Link Point on the Relaxation Behaviors of Transesterification Vitrimers,” Macromolecules 55, no. 15 (2022): 6661–6670, 10.1021/acs.macromol.2c00560.

[29] L. Li , X. Chen , K. Jin , and J. M. Torkelson , “Vitrimers Designed both To Strongly Suppress Creep and To Recover Original Cross‐Link Density After Reprocessing: Quantitative Theory and Experiments,” Macromolecules 51, no. 15 (2018): 5537–5546, 10.1021/acs.macromol.8b00922.

[30] J. L. Self , N. D. Dolinski , M. S. Zayas , J. Read de Alaniz , and C. M. Bates , “Brønsted‐Acid‐Catalyzed Exchange in Polyester Dynamic Covalent Networks,” ACS Macro Letters 7, no. 7 (2018): 817–821, 10.1021/acsmacrolett.8b00370. 35650774

[31] F. Cuminet , S. Caillol , É. Dantras , É. Leclerc , and V. Ladmiral , “Neighboring Group Participation and Internal Catalysis Effects on Exchangeable Covalent Bonds: Application to the Thriving Field of Vitrimer Chemistry,” Macromolecules 54, no. 9 (2021): 3927–3961, 10.1021/acs.macromol.0c02706.

[32] N. Bouillon , J. Pascault , and L. Tighzert , “Influence of different imidazole catalysts on epoxy‐anhydride copolymerization and on their network properties,” Journal of Applied Polymer Science 38, no. 11 (1989): 2103–2113, 10.1002/app.1989.070381112.

[33] M. A. Corcuera , I. Mondragon , C. C. Riccardi , and R. J. J. Williams , “Polymer Networks Derived From the Anhydride Curing of Tetraepoxides,” Journal of Applied Polymer Science 64, no. 1 (1997): 157–166, 10.1002/(SICI)1097-4628(19970404)64:1<157::AID-APP14>3.0.CO;2-1.

