# Supplementary material for: Recycling Thermoset Systems by Vitrimerization Using Solid‐State Shear Extrusion‐ A Feasibility Study
Source: Glob Chall. 2025 Dec 4;10(1):e00417. doi: 10.1002/gch2.202500417 (PMC12776006; doi:10.1002/gch2.202500417)
Supplement: Supplementary file 1 — Supporting file: gch270075‐sup‐0001‐SuppMat.docx [file GCH2-10-e00417-s001.docx]

**Supporting Information**

**Recycling Thermoset Systems by Vitrimerization Using Solid-State Shear Extrusion - A Feasibility Study**

**Amin Jamei-Oskouei, Majid Mehrabi-Mazidi, Ica Manas-Zloczower^*^**

Department of Macromolecular Science and Engineering, Case Western Reserve University, Cleveland, OH 44106, USA

**Corresponding Author:**

***Ica Manas-Zloczower, E-mail:** [**ixm@case.edu**](mailto:ixm@case.edu)





Figure S1. FTIR spectra for the initial epoxy sample and vitrimerized samples via ball-milling (BM-Epoxy-vitrimer) and solid-state shear extrusion (SSSE-Epoxy-vitrimer).


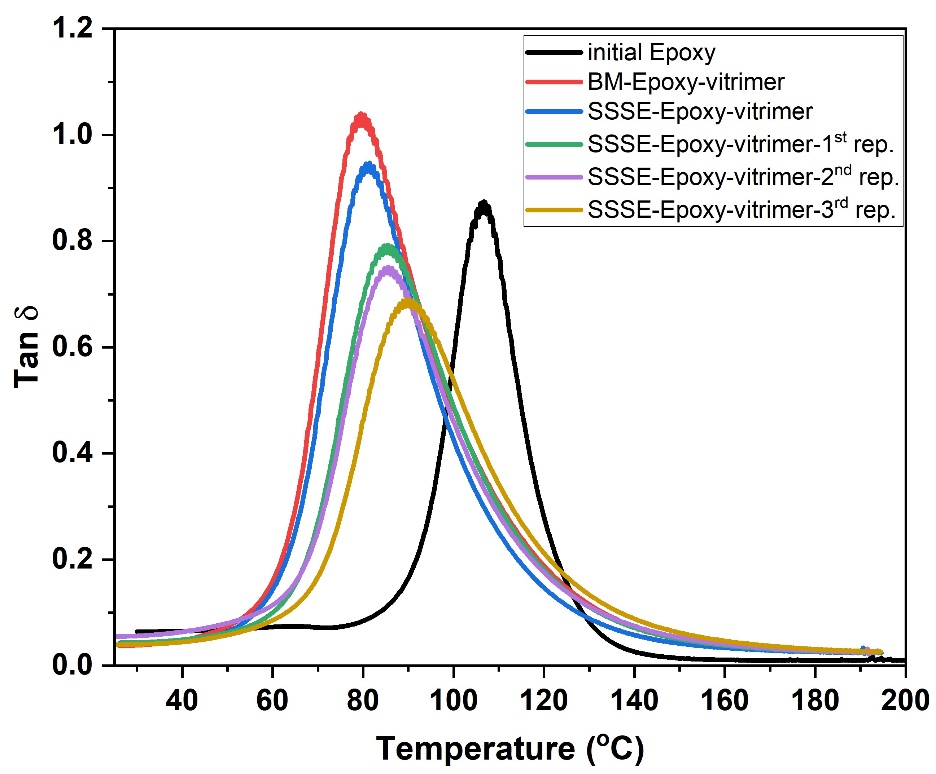


Figure S2. Temperature dependence of loss tangent for initial epoxy, BM-epoxy-vitrimer, SSSE-Epoxy-vitrimer, and reprocessed SSSE vitrimers obtained after three reprocessing cycles.

**Cross-linking density calculation.** The effective crosslinking densities (ρ) of initial and vitrimerized epoxies were calculated according to the rubber elasticity theory using the following equation (1):^6^

$\rho=\frac{E^{'}}{3RT}$ (1)

Where E′ is the storage modulus at the plateau region, R is the gas constant, and T is the absolute temperature at 180 °C.

Table S1. Crosslink density calculation by using rubber elasticity theory and rubbery plateau in DMA tests.

| Samples | Initial Epoxy | BM-Epoxy-vitrimer | SSSE-Epoxy-vitrimer | SSSE-Epoxy-vitrimer-Reprocessed | | | | |
| --- | --- | --- | --- | --- | --- | --- | --- | --- |
|  |  |  |  | 1^st^ | 2^nd^ | | | 3^rd^ |
| Crosslink density (ρ) [mol/m^3^] | 1275 ± 84 | 313 ± 23 | 407 ± 67 | 575 ± 4 | | 730 ± 22 | 824 ±13 | |

**Stress relaxation time fitting**

Figures S3 and S4 show stress relaxation tests for vitrimerized systems using ball milling (BM) and solid-state shear extrusion (SSSE) at various temperatures. The stress relaxation fittings were conducted in accordance with our previous work and that of others.^1–5^ and the fitting parameters are presented in Tables S2 and S3. The Kohlrausch-Williams-Watts (KWW) stretched exponential decay is given below (2):

$\frac{E(t)}{E_{0}}=exp\left\{ {-\left( \frac{t}{\tau^{*}} \right)}^{\beta} \right\}$ (2)

where ${E(t)}/{E_{0}}$ is the normalized relaxation modulus at time t, $\tau^{*}$ is the characteristic relaxation time, and the exponent $\beta$ controls the shape of the stretched exponential decay between 0.5 and 1.





Figure S3. Normalized relaxation modulus over time curves at different temperatures showing the stress relaxation of epoxy vitrimerized by ball milling. Three independent specimens were tested at each temperature to demonstrate replicability





Figure S4. Normalized relaxation modulus over time curves at different temperatures showing the stress relaxation of epoxy vitrimerized by solid-state shear extrusion. Three independent specimens were tested at each temperature to demonstrate replicability.

Table S2. Extracted data from fitting the KWW stretched exponential decay equation on stress relaxation curves of vitrimerized epoxy using ball milling at different temperatures.

| Temperature (C) | | τ* (s) | β | <τ> (s) | Adjusted R^2^ |
| --- | --- | --- | --- | --- | --- |
| 200 | 200-1 | 410.86 ± 6.06 | 0.91 ± 0.02 | 429.73 ± 6.33 | 0.99 |
|  | 200-2 | 367.52 ± 6.56 | 0.97 ± 0.02 | 371.94 ± 6.66 | 0.99 |
|  | 200-3 | 337.67 ± 6.14 | 0.94 ± 0.02 | 347.70 ± 6.35 | 0.99 |
| 220 | 220-1 | 102.38 ± 0.87 | 0.85 ± 0.01 | 111.38 ± 0.97 | 0.99 |
|  | 220-2 | 155.93 ± 1.58 | 0.86 ± 0.01 | 168.50 ± 1.74 | 0.99 |
|  | 220-3 | 184.14 ± 1.15 | 0.86 ± 0.01 | 198.75 ± 1.26 | 0.99 |
| 250 | 250-1 | 63.99 ± 0.81 | 0.84 ± 0.01 | 70.14 ± 0.91 | 0.99 |
|  | 250-2 | 58.51 ± 1.21 | 0.86 ± 0.02 | 63.73 ± 1.33 | 0.99 |
|  | 250-3 | 71.97 ± 1.34 | 0.81 ± 0.02 | 81.58 ± 1.54 | 0.99 |
| 280 | 280-1 | 46.79 ± 1.36 | 0.79 ± 0.03 | 53.50 ± 1.58 | 0.99 |
|  | 280-2 | 37.38 ± 1.00 | 0.87 ± 0.03 | 39.69 ± 1.10 | 0.99 |
|  | 280-3 | 27.17 ± 0.70 | 0.88 ± 0.03 | 28.77 ± 0.78 | 0.99 |
| Apparent flow activation energy (KJ/mol) | | 66.25 ± 3.43 | | | 0.99 |

Table S3. Extracted data from fitting the KWW stretched exponential decay equation on stress relaxation curves of vitrimerized epoxy using solid-state shear extrusion at different temperatures.

| Temperature (C) | | τ* (s) | β | <τ> (s) | Adjusted R^2^ |
| --- | --- | --- | --- | --- | --- |
| 200 | 200-1 | 489.00 ± 11.41 | 0.67 ± 0.01 | 646.65 ± 13.96 | 0.99 |
|  | 200-2 | 396.26 ± 9.36 | 0.68 ± 0.01 | 515.75 ± 11.43 | 0.99 |
|  | 200-3 | 406.58 ± 8.79 | 0.72 ± 0.01 | 501.92 ± 10.16 | 0.99 |
| 220 | 220-1 | 539.15 ± 12.96 | 0.66 ± 0.01 | 724.43 ± 15.97 | 0.99 |
|  | 220-2 | 234.77 ± 1.20 | 0.86 ± 0.00 | 253.83 ± 1.32 | 0.99 |
|  | 220-3 | 208.18 ± 1.13 | 0.85 ± 0.01 | 226.29 ± 1.25 | 0.99 |
| 250 | 250-1 | 146.89 ± 3.10 | 0.73 ± 0.01 | 178.95 ± 4.13 | 0.99 |
|  | 250-2 | 67.84 ± 1.35 | 0.81 ± 0.02 | 77.04 ± 1.55 | 0.99 |
|  | 250-3 | 58.99 ± 1.22 | 0.85 ± 0.02 | 64.19 ± 1.35 | 0.99 |
| 280 | 280-1 | 35.51 ± 0.83 | 0.83 ± 0.02 | 39.23 ± 1.20 | 0.99 |
|  | 280-2 | 43.04 ± 1.18 | 0.87 ± 0.03 | 46.13 ± 1.29 | 0.99 |
|  | 280-3 | 51.26 ± 1.77 | 0.83 ± 0.03 | 56.33 ± 1.99 | 0.99 |
| Apparent flow activation energy (KJ/mol) | | 67.76 ± 4.02 | | | 0.99 |


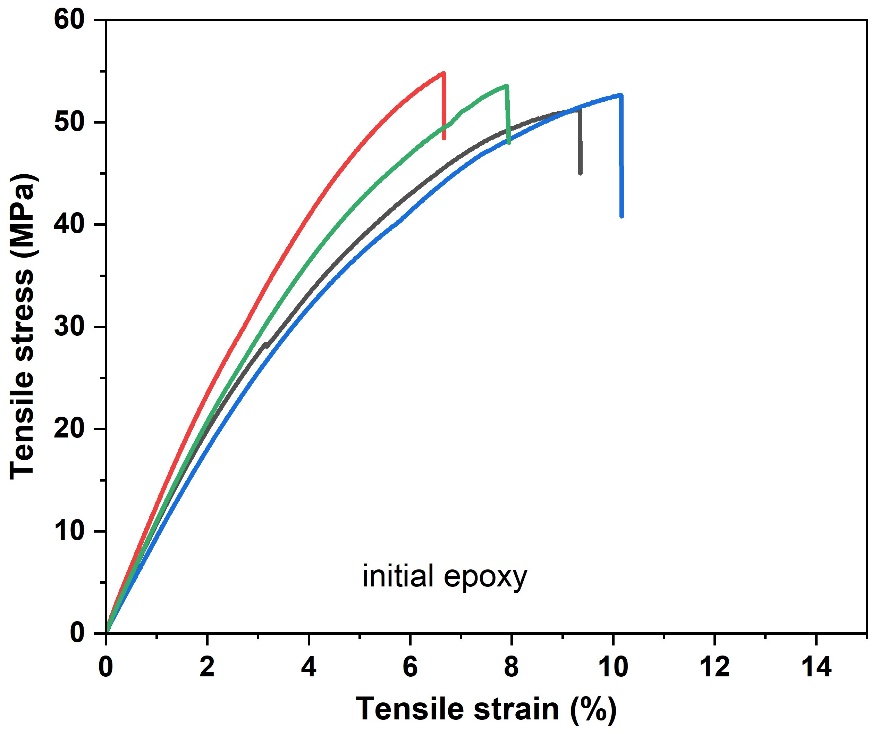


Fig. S5: Tensile stress-strain curves obtained for initial epoxy.


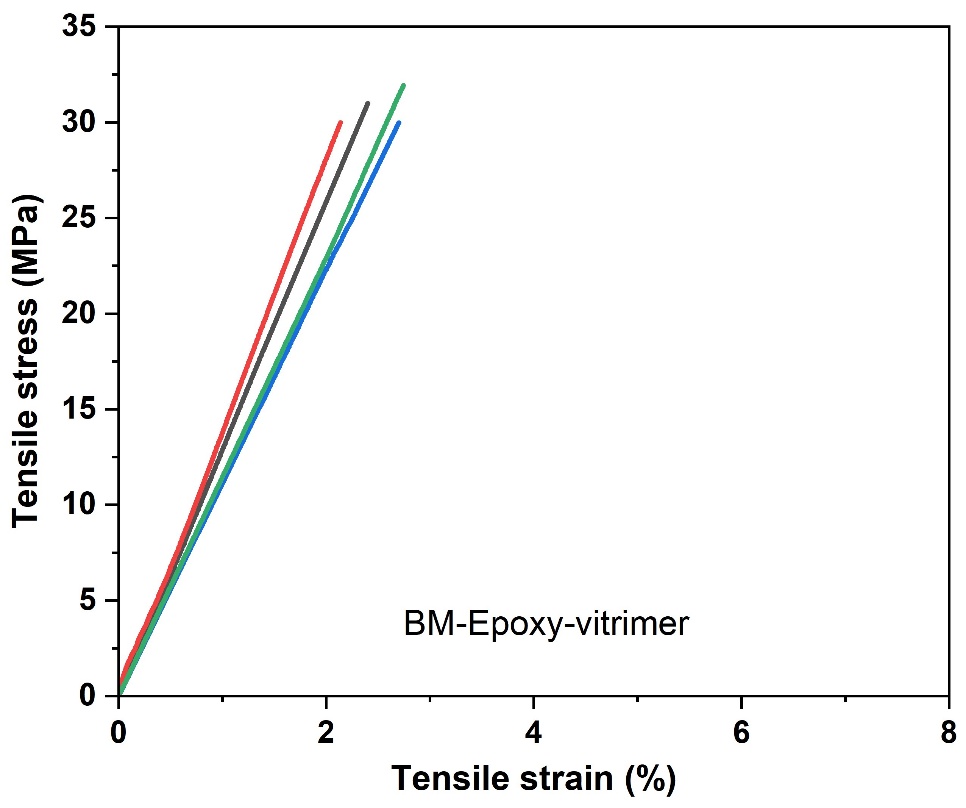


Fig. S6: Tensile stress-strain curves obtained for BM-epoxy vitrimer.


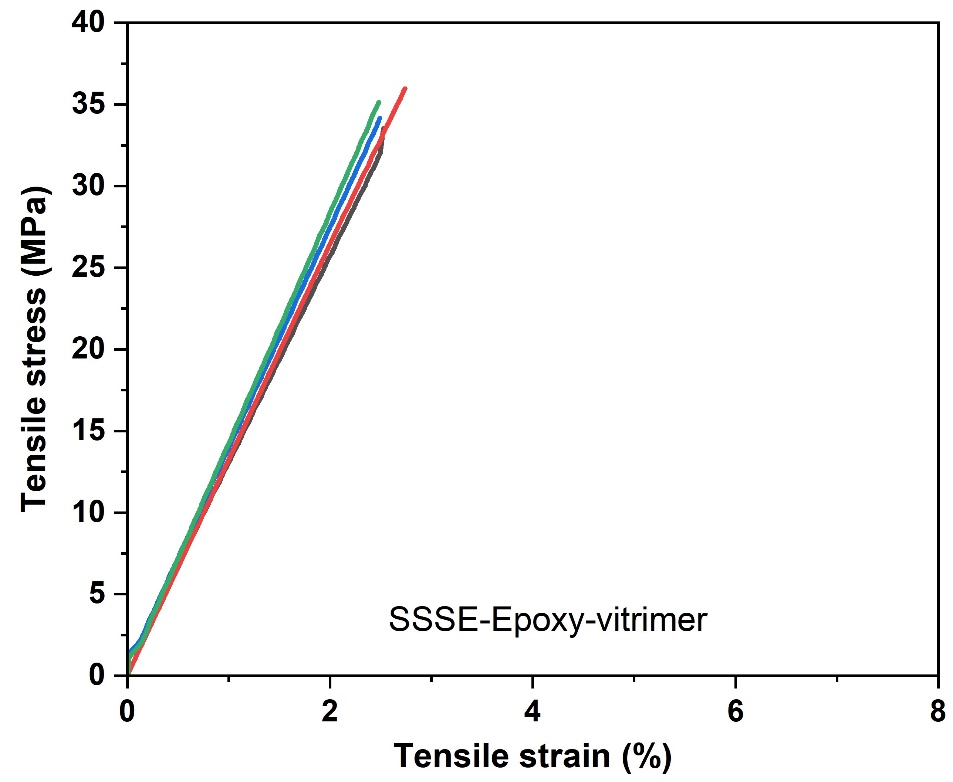


Fig. S7: Tensile stress-strain curves obtained for SSSE epoxy vitrimer.


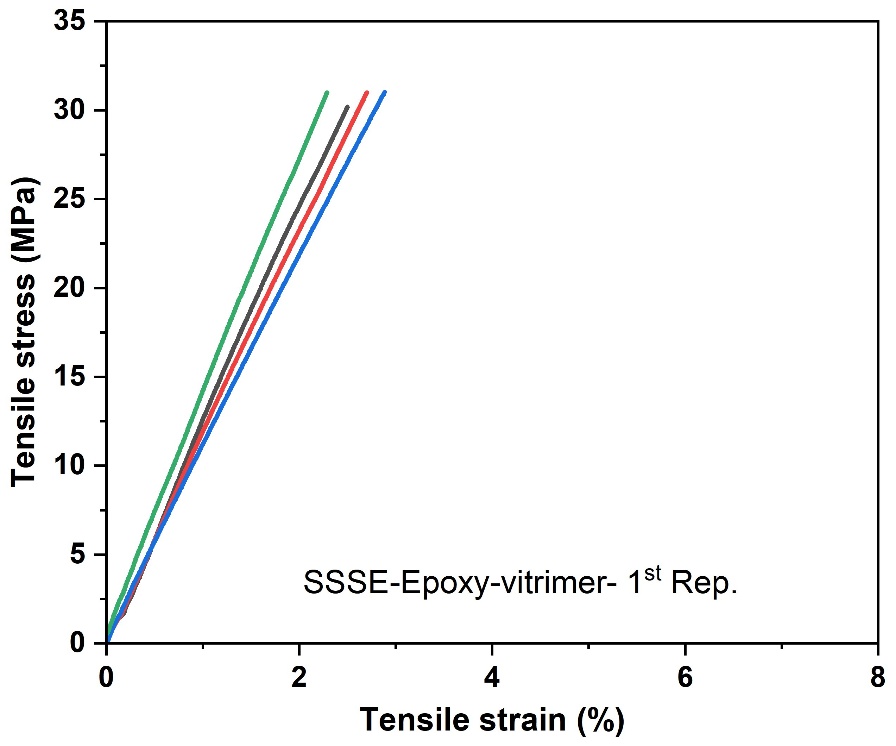


Fig. S8: Tensile stress-strain curves obtained for reprocessed SSSE epoxy vitrimer.


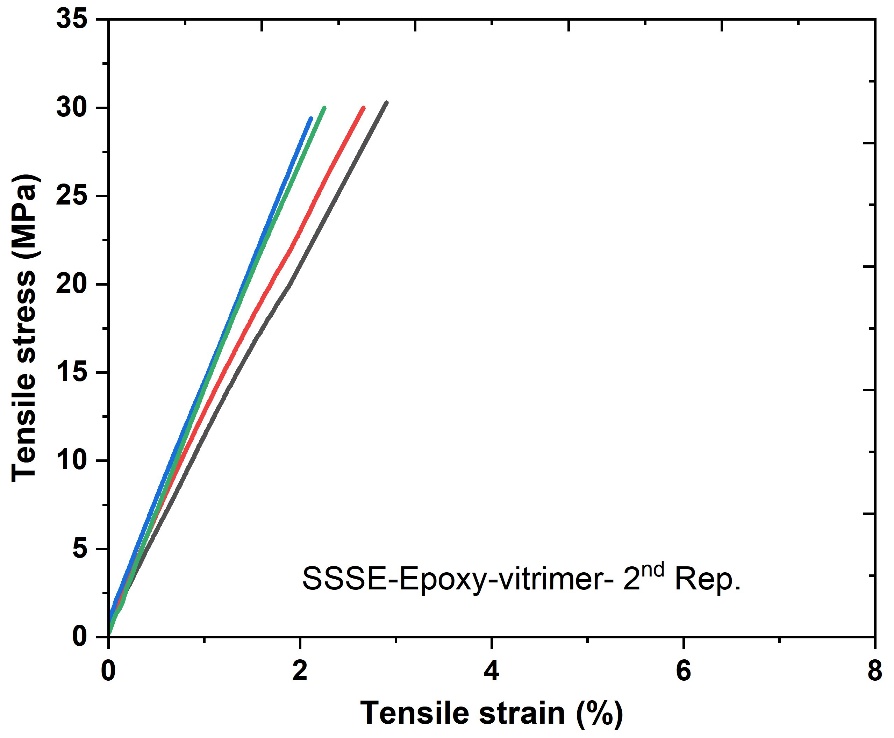


Fig. S9: Tensile stress-strain curves obtained for reprocessed SSSE epoxy vitrimer.


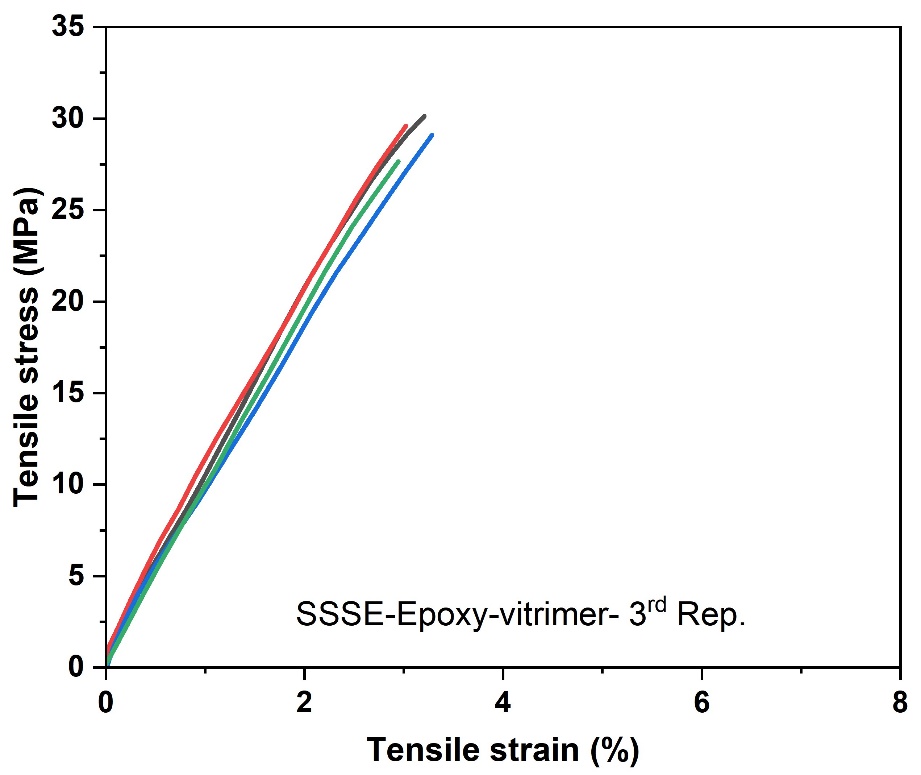


Fig. S10: Tensile stress-strain curves obtained for reprocessed SSSE epoxy vitrimer.


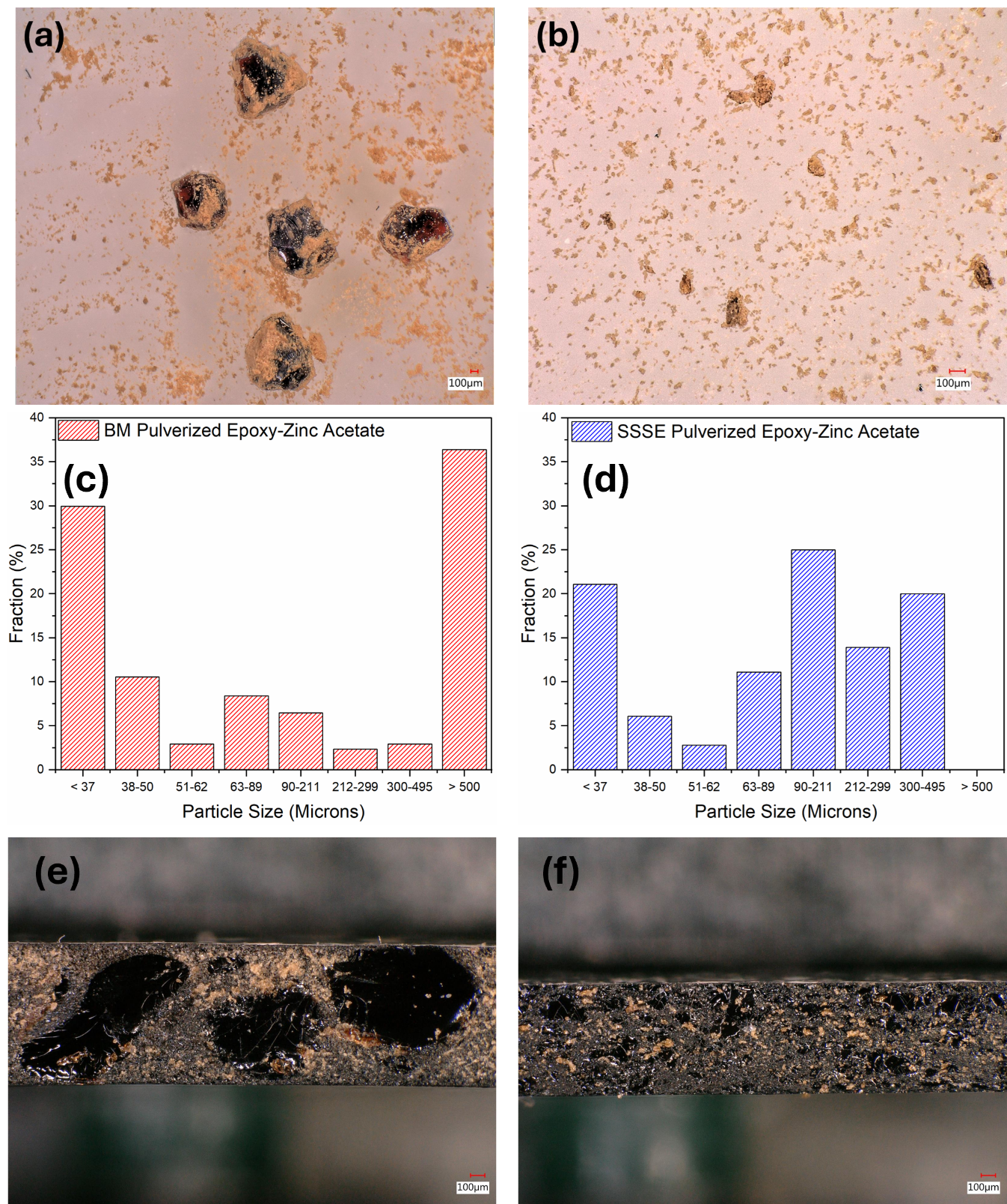


Figure S11. Optical micrograph of pulverized powder mixtures after (a) ball-milling, (b) solid-state shear extrusion, and size distribution measurements by sieving of (c) ball-milled pulverized epoxy and zinc acetate, (d) solid-state shear extrusion pulverization of epoxy and zinc acetate. (e, f) Optical images taken from the cross-section of molded tensile specimens for BM and SSSE vitrimers, respectively.





Figure S12.TGA for initial epoxy, BM-Epoxy-vitrimer, SSSE-Epoxy-vitrimer, and SSSE-Epoxy-vitrimer-Reprocessed.

**References**

(1) Li, L.; Chen, X.; Jin, K.; Torkelson, J. M. Vitrimers Designed Both To Strongly Suppress Creep and To Recover Original Cross-Link Density after Reprocessing: Quantitative Theory and Experiments. *Macromolecules* **2018**, *51* (15), 5537–5546. https://doi.org/10.1021/acs.macromol.8b00922.

(2) Cuminet, F.; Berne, D.; Lemouzy, S.; Dantras, É.; Joly-Duhamel, C.; Caillol, S.; Leclerc, É.; Ladmiral, V. Catalyst-Free Transesterification Vitrimers: Activation via α-Difluoroesters. *Polymer Chemistry* **2022**, *13* (18), 2651–2658. https://doi.org/10.1039/D2PY00124A.

(3) Isogai, T.; Hayashi, M. Critical Effects of Branch Numbers at the Cross-Link Point on the Relaxation Behaviors of Transesterification Vitrimers. *Macromolecules* **2022**, *55* (15), 6661–6670. https://doi.org/10.1021/acs.macromol.2c00560.

(4) Bandegi, A.; Gray, T. G.; Mitchell, S.; Jamei Oskouei, A.; Sing, M. K.; Kennedy, J.; Miller McLoughlin, K.; Manas-Zloczower, I. Vitrimerization of Crosslinked Elastomers: A Mechanochemical Approach for Recycling Thermoset Polymers. *Mater. Adv.* **2023**, *4* (12), 2648–2658. https://doi.org/10.1039/D3MA00098B.

(5) Oskouei, A. J.; Mao, E.; G. Gray, T.; Bandegi, A.; Mitchell, S.; K. Sing, M.; Kennedy, J.; McLoughlin, K. M.; Manas-Zloczower, I. Vitrimerization of Crosslinked Poly(Ethylene-Vinyl Acetate): The Effect of Catalysts. *RSC Applied Polymers* **2024**, *2* (5), 905–913. https://doi.org/10.1039/D4LP00112E.

(6) Yue, L.; Guo, H.; Kennedy, A.; Patel, A.; Gong, X.; Ju, T.; Gray, T.; Manas-Zloczower, I. Vitrimerization: Converting Thermoset Polymers into Vitrimers. *ACS Macro Lett.* **2020**, *9* (6), 836–842. https://doi.org/10.1021/acsmacrolett.0c00299.
